# Domestic Correspondence

**Published:** 1889-11

**Authors:** 


### Domestic Correspondence.

To the Editor:

We noticed an editorial article in the October number of the *Dental Review*, written in reply to a letter published in the same number of that journal from the pen of Dr. Allport, which, to our mind, needs a few words of comment.

The editor of the *Review* could not have done more wisely than to have accepted the suggestion for a "Memorial" meeting of the American Dental Association in 1892, and thrown what influence he might have in that direction.

But for reasons best known to himself, he thought best to put his editorial veto upon the suggestion, and upon the best interests of the National Association of the dental profession of America,—the society which has elevated him to the next highest position in its power to give,—and turn the influence of his journal towards an International Dental Congress, which, I was about to say, nobody in America wants. This statement, however, would not be correct, for there are a few who seem very anxious for it.

The suggestion of a "memorial meeting," by Dr. Allport, was called out by a previous editorial in the *Review* for September, which lamented the evident lack of interest in the affairs of the Association; the object being to set on foot a plan for stimulating the flagging interest, and placing the Association again in its old field as the foremost society of its kind in our land.

If an International Dental Congress is to be called, the proper parties to do it are a majority of the members of the American and Southern Dental Associations.

It has been customary in like gatherings for the National Association to extend the invitation, appoint the committees, and make all the arrangements.

At the meeting of the American Dental Association, held in August last, this question was made a *special order of business*, in order that there might be a free discussion of the whole matter. The whole question, however, went by default, as nobody seemed to feel that such a Congress was needed in America. It was well enough to hold a Congress in France, and doubtless it was productive of great good.

Such would have been the case if a meeting could have been held in this country thirty or forty years ago, before the profession was so thoroughly organized as it is to-day. At the present, the profession of America does not need it, as we are well organized and are recognized as a department of medicine, and identified with the International Medical Congress as one of its most important departments.

We were sorry also to see the spirit manifested by the editor of the *Review* in speaking of the members of the Association who were also members of the dental section of the American Medical Association. He must have been a little out of temper, certainly, when he penned the following paragraph: "First and foremost is the evident tendency of the members of the dental section of the American Medical Association to control the American Dental Association and direct its policy. . . . The efforts spent in this direction, if they had been directed to the more profitable channels of preparing papers and presentation of scientific work in the meetings of the Association, would not now call for a 'memorial' meeting of the Association in 1892."

In the first part of this paragraph he unintentionally pays the members of the "dental section" a very high compliment, for, if our memory serves us correctly, there were but six *active* members of this section in attendance at the Saratoga meeting of the American Dental Association, who, by the way, are all loyal members of the last-named society. We were not aware that these six, or any other number of the dental section, had such great influence in the Association, or that they had ever tried unlawfully to "control" or "direct the policy" of the Association. It is true, they have tried to exert an individual influence for good, as it seemed to them, but never, to our knowledge, have they been found upon that side which, for selfish reasons, would attempt to destroy or cripple the old and honored national society, and upon its ruined or dismembered body to build a rival institution or an International Dental Congress. Such an institution could be of no permanent value to the profession of this country, and would only tend to weaken the existing societies.

In the latter part of the paragraph quoted the editor of the *Review* would convey the idea that the members of the dental section did nothing to advance the scientific interest of the meeting. By way of refreshing his memory, we will state that two of the six just mentioned (Drs. Talbot and Sudduth) read papers of scientific interest to the Association, and that all but one took an active part in the discussions of scientific and other matters brought before the society; and four of the six before mentioned read papers in the dental section of the American Medical Association; while, on the other hand, the editor of the *Dental Review* reserved his strength for a paper which he read, not in the American Dental Association, but in the International Dental Congress, held a little later in Paris. "Consistency thou art a jewel."

JOHN S. MARSHALL.

CHICAGO, ILL.

---

TO THE EDITOR:

Enclosed please find a few practical points that may be of use to your readers. As time passes, teeth that have lost their pulps seem to lose also, in an increasing degree, the elasticity of dentine which enables them to withstand the normal strain of mastication. The result is that many pulpless teeth are broken by the force exerted against them through their antagonists in the opposite jaw. How many wrecks of bicuspids and badly-broken molars might have been guarded from such a deplorable condition as we find them in, when their owners bring them for repairs after the break, if their lingual cusps had been ground off, so that the force exerted against them could not have been expended in such a direction as to produce such disastrous consequences! The splitting and breaking of the inner sides of pulpless bicuspids and molars, which so generally extends some distance under the gum, is an entirely preventable accident. It requires a strong conviction and firm purpose in preventive treatment to mutilate a tooth by grinding off its inner cusps, but when the pulp has been lost, it seems to be a clear case of the need to choose between evils. Either heroically remove that portion of the tooth against which the force can be exerted to split off the inner fourth or half, or else permit your patient to run the risk of loss you can but poorly repair, with the knowledge that whenever the occlusion is anything like normal, such damage is almost certain to result, sooner or later. The grindstone need remove only one-fourth or less of the amount of tooth-structure that will probably be removed by the break, to make the remainder perfectly safe from such ill-fated liability.

*Retaining Pits.*—The term seems to be a misnomer. Few fillings are or could be retained permanently by pits. The general shape of a cavity must be such as to retain a filling, or it will not remain. The drill-holes made in the dentine, called "retaining pits," can at best only retain the part of the filling anchored in them, while more is added in the process of construction; and for this purpose, even when a cavity is well prepared, they may be reduced to a minimum. One shallow pit in one corner will almost always serve the purpose, and even this may be frequently dispensed with. The practice of drilling pits in the bottom or sides of cavities, to start or retain fillings, is undoubtedly responsible for the death of many pulps. The latter do not tolerate the near approach of a conducting material, when that proximity is suddenly thrust upon them in drilling into sound dentine, for instance, in nearly so great a degree as when the approach of irritating agents has been more gradual, as in the slower advance of decay. Yet in the latter case it is a common and wise practice to interpose some non-irritating, non-conducting medium, where cavities are deep; but if it so happens that there appears to be a difficulty in starting or making a filling, it is not uncommon, it appears, to not only dispense with the non-conducting medium under the filling, but in addition to immensely increase the danger to the pulp by drilling one or more holes into the sound dentine, into which one of the best of conductors is packed. Fortunately, the increasing use and appreciation of non-cohesive gold is making the dangerous and seductive retaining pit less important in the process of constructing gold fillings.

J. Morgan Howe.

New York City.

---

To the Editor:

As the saving of time is an object to most dentists, I will describe a process of soldering small pieces of gold work, which, though not new to all, may be of service to many. If you have a plate with two or three teeth which you wish to attach by means of solder, back the teeth and fasten in position by means of hard wax; then take moulding-sand and wet it thoroughly, until it is of the consistency of soft putty; place this on your soldering block, press the plate into it, and bring the sand well up around the teeth. Now take your blow-pipe and throw a broad, gentle flame around the outer edges of the sand, taking care not to let the flame touch the plate or teeth until the water is driven off and the wax begins to blaze; then direct the flame upon the wax and burn it off. Scrape well the parts upon which you wish the solder to flow; then place on the solder and borax, and proceed as usual. Partly fill a sauce-pan with water, and place it over a gas- or oil-stove, and when it boils hold the case, wrapped (investment and all) in a cloth, over the steam for half a minute close to the water; then drop it in, remove and take out the piece. Clasps and small regulating pieces are held together and soldered by this process very quickly.

I have yet to crack my first tooth by soldering in this manner, which, I think, is due to the fact that the expansion by steam heat is more uniform than by dry.

Sand which has been used for moulding purposes is dangerous to use, as particles of zinc or lead may be present, and thus become alloyed with the gold. J. Bond Littig.

New York City.

---

To the Editor:

Since the publication of my paper on "The Bonwill Method of Packing Gold Foil," in the September issue of your journal, I have been made conscious, through inquiries, as to certain details of the method which I tried to explain, that my description in the paper has failed to make sufficiently clear one item of especial importance,—viz., the character of the surface of the plugger-points used. What I said in the paper with reference to this was as follows: "The use of this method demands that the plugger-points, whatever their size, angle, or general form may be, shall have slightly convex faces, with extremely shallow serrations and rounded edges, *so that they shall not tear the gold.*" This statement should have been made fuller and more explicit. The points which were originally furnished to me by Dr. Bonwill were serrated about as finely as those of the Varney set of instruments, when they came from the hands of the maker; but before sending them to me, Dr. Bonwill, as I afterwards learned, had removed nearly all of the serrated surface by rubbing the points over an Arkansas stone, so that, when they came into my possession, while the lines of the original serrations were distinctly visible, the surface of the plugging-point was nearly smooth. Subsequent use has rendered them entirely so, and they have never been recut. All the points which I have since had made for my own use have gone through the same treatment by having the sharpness of the serrations obliterated by the Arkansas stone. It is, of course, obvious that any serration, in the ordinary sense, would be fatal to the efficiency of a plugging-point when used for packing gold by the method under consideration, the whole spirit of it being dependent upon the sliding or planishing movement of the tool as described. Hence, the older and more worn the plugging-surface of the point becomes, the greater its efficiency within reasonable limits.

I have recently learned that Dr. Bonwill uses in his own work uncut points, absolutely devoid of serrations, which are smooth like burnisher-points. This fact I was not aware of at the time my paper was read. Nor have I had any experience with the use of such points; but, as a matter of fact, those which are in daily use in my office are probably as smooth and as devoid of serration—as a result of the usual wear and tear—as those which have never been serrated. I trust the foregoing explanation may help to render clear what I had to say in reference to a method which has made for me the packing of gold foil in teeth a pleasure instead of a laborious and fatiguing process.

EDWARD C. KIRK.

PHILADELPHIA.

